# Discriminating between Homogeneous (AC-1) and Dense Fine Speckled (AC-2) Antinuclear Antibody Patterns: Re-Evaluation of Immunofluorescence Imaging

**DOI:** 10.3390/biomedicines11113027

**Published:** 2023-11-11

**Authors:** Han-Hua Yu, Pao-Feng Hsieh, Szu-Wei Huang, Tien-Ming Chan, Pao-Lien Tai, Shih-Ting Yang, Kuang-Hui Yu

**Affiliations:** 1 Division of Rheumatology, Allergy and Immunology, Linkou Chang Gung Memorial Hospital, Taoyuan 333, Taiwan; 2 College of Medicine, Chang Gung University, Taoyuan 333, Taiwan

**Keywords:** antinuclear antibody, ANA, homogeneous, dense fine speckled, DFS70, immunofluorescence

## Abstract

Antinuclear antibodies (ANAs) are essential diagnostic markers in systemic autoimmune rheumatic diseases. Among the 30 ANA patterns, homogeneous (AC-1) and dense fine speckled (AC-2) should be focused on owing to their somewhat indistinct presentation in immunofluorescence imaging and distinct correlation with clinical conditions. This study aimed to develop a flowchart to guide discrimination between AC-1 and AC-2 patterns and to re-evaluate ANA samples according to this flowchart to verify its detection ability. We re-evaluated immunofluorescence imaging of 62 ANA blood samples simultaneously subjected to solid-phase assays for autoantibodies against dsDNA, nucleosomes, histones, and DFS70. The results showed statistically significant odd ratios (ORs) of detection of anti-DFS70 using AC-2 after re-evaluation of total samples (OR 101.9, 95% CI 11.7–886.4, *p*-value < 0.001) and subgroup analysis of patients’ samples (OR 53.8, 95% CI 5.9–493.6, *p*-value < 0.001). The OR of anti-nucleosome/histone/dsDNA detection using AC-1 in re-evaluated data increased to 5.43 (95% CI 1.00–29.61, *p*-value = 0.05). In the analysis of specific autoantibodies, more than half of the samples with an AC-2 pattern (54.2%) had specific autoantibodies other than anti-DFS70. We conclude that the flowchart for discriminating between AC-1 and AC-2 ANA patterns in this study is a viable practical guide for other laboratories when encountering equivocal ANA results.

## 1. Introduction

Antinuclear antibodies (ANAs) are recognized as essential diagnostic markers in systemic autoimmune rheumatic diseases (SARDs), such as systemic lupus erythematosus (SLE), systemic sclerosis, idiopathic inflammatory myopathy, primary biliary cholangitis, Sjögren’s syndrome, and mixed connective tissue disease [1,2]. Indirect immunofluorescence assays (IIFAs) using human epithelial type 2 (HEp-2) cells as substrate are the gold standard for the detection of ANAs [2,3]. Thirty different HEp-2 cell IIFA patterns (1 negative ANA pattern denoted as AC-0, and 29 positive ANA patterns denoted from AC-1 to AC-29) were established in the International Consensus on ANA Patterns (ICAP) [1,3]. The 29 positive ANA patterns are further categorized as nuclear, cytoplasmic, and mitotic patterns: AC-1 to AC-14 and AC-29 are classified as nuclear HEp-2 IIFA patterns, AC-15 to AC-23 are classified as cytoplasmic HEp-2 IIFA patterns, and AC-24 to AC-28 are classified as mitotic HEp-2 IIFA patterns. ANA patterns and their clinical relevance has been widely investigated. SLE is associated with AC-1, AC-4, AC-5, AC-13, AC-19, AC-24, and AC-26. Systemic sclerosis is associated with AC-3, AC-4, AC-5, AC-8, AC-9, AC-10, AC-13, AC-21, AC-24, AC-26, and AC-29. Idiopathic inflammatory myopathy is associated with AC-4, AC-5, AC-6, AC-8, AC-13, AC-19, and AC-20. Sjögren’s syndrome is associated with AC-4, AC-10, AC-14, AC-21, and AC-26. The updated ANA classification and related information can be found on the official website of the ICAP (anapatterns.org). However, ANA testing has high interobserver variability and difficult standardization; an ANA result based on IIFA HEp-2 substrates may mislead the physician in some cases and has to be interpreted within the clinical situation [4]. In addition to the variability of positive ANA results, negative results of ANA assays may also yield positive autoantibodies to extractable nuclear antigens, such as SSA, SSB, RNP, Sm, Scl-70, and Jo-1 [5]. As a result, follow-up testing with solid-phase assays, such as ELISA, may be essential in clinical practice after performing ANA assays [2,4].

Among the 30 ANA patterns, homogeneous (AC-1) and dense fine speckled (AC-2) patterns should be focused on owing to their somewhat indistinct presentation in immunofluorescence imaging and distinct correlation with clinical conditions. According to the ICAP statement, the AC-1 pattern, characterized by a homogeneous nucleoplasm during interphase with an intensely stained chromatin mass in a homogeneous hyaline fashion in mitotic cells, is associated with autoantibodies to dsDNA, nucleosomes, and histones, which are mostly related to SLE [1,6,7]. In contrast, the AC-2 pattern, characterized by a heterogenous speckled nucleoplasm during interphase with a strongly speckled pattern and some coarse speckles standing out of the metaphase plate, is associated with autoantibodies against the dense fine speckled protein of 70 kD (DFS70) (also known as lens epithelium-derived growth factor protein of 75 kD, LEDGF/p75), which is negatively associated with SARDs [1,6,8]. In clinical laboratories, recognition of the AC-2 pattern in IIFA testing is very discretionary and subjective, even for expert staff [9]. Although a sufficiently detailed description of the AC-2 pattern has been provided by ICAP, it is still quite challenging to detect the AC-2 pattern accurately, especially for technologists unfamiliar with this pattern or for automated detection devices. A monospecific AC-2 pattern is often confused with the AC-1 pattern, and the AC-2 pattern is very difficult to identify when concomitant with other ANA patterns [10,11]. The clinical relevance of the AC-2 pattern, which can help exclude autoimmune diseases, becomes evident only when it is confirmed to be monospecific for the anti-DFS70 autoantibody [12].

The present study aimed to analyze serum samples with AC-1 and AC-2 patterns subjected to solid-phase assays for related autoantibodies and to develop a guide flowchart to help discriminate between AC-1 and AC-2 patterns. We then re-evaluated the included ANA samples according to our developed flowchart and verified its detection ability.

## 2. Materials and Methods

### 2.1. Samples

This retrospective study included immunofluorescence imaging of 62 ANA blood samples, simultaneously subjected to solid-phase assays for autoantibodies against dsDNA, nucleosomes, histones, and DFS70, from the Immunology Laboratory of Linkou Chang Gung Memorial Hospital (CGMH) between February and May 2023. The 62 ANA samples included 19 subjects for health examination and 43 patients. Among the 19 subjects for health examination, there were 10 AC-1 and 9 AC-2 ANA patterns. Among the 43 patients, there were 10 AC-1, 12 AC-2, and 21 equivocal AC-1/AC-2 ANA patterns.

All blood samples were sent to the Immunology Laboratory of Linkou CGMH, which is certified by the College of American Pathologists. External quality control for the laboratory data was achieved through participation in the international program of the College of American Pathologists and the National Quality Control Program, administered by the Taiwanese government [13]. Sera from subjects for health examination and from patients were subjected to AESKUSLIDES ANA-HEp-2 (AESKU.DIAGNOSTICS, Wendelsheim, Germany) for ANA testing, and to EUROLINE ANA Profile 23 or EUROLINE ANA Profile 3 plus DFS70 (EUROIMMUN, Lübeck, Germany) for solid-phase assays to confirm the specific autoantibodies.

This study was approved by the Institutional Review Board of Chang Gung Medical Foundation (IRB No.: 202301274B0).

### 2.2. ANA IIFA

We used AESKUSLIDES ANA-HEp-2 (AESKU.DIAGNOSTICS, Wendelsheim, Germany) to perform ANA IIFA. Following the manufacturer’s instructions, the testing procedure was performed.

1. First incubation: Pipette an adequate volume of control samples and diluted serum samples (1:80 with sample buffer) into each well of slides. Incubate slides for 30 min ± 10 min at room temperature (20–26 °C) in the moist incubator tray.
2. Washing: Remove slides from incubator tray and rinse briefly with wash buffer using a squeeze wash bottle. Wash slides 2 × 5 min with wash buffer in a slide staining dish.
3. Second incubation: Return slides to incubator tray and cover each well with an adequate volume of FITC-conjugate (fluorescein labelled anti-human IgG). Incubate slides for 30 min ± 10 min at room temperature in the dark.
4. Washing: Wash as described above.
5. Mounting medium: Add an adequate volume of mounting medium along midline of each slide.
6. Reading: Read slides at 400–800 × total magnification with a fluorescent microscope.

The ANA endpoint titers of the included sample reports ranged from 1:80 to >1:1280 according to the fluorescence intensity. Considering the ANA endpoint titer of 1:80 as the normal limit in some studies [14,15], subgroup analysis to exclude samples with an ANA endpoint titer of 1:80 was also performed.

### 2.3. Solid-Phase Assay

We used EUROLINE ANA Profile 23 or EUROLINE ANA Profile 3 plus DFS70 (EUROIMMUN, Lübeck, Germany) for solid-phase assays to confirm specific autoantibodies, including those against dsDNA, nucleosomes, and histones, that are associated with AC-1, and those against DFS70, which is associated with AC-2. Following the manufacturer’s instructions, the assay procedure was performed.

1. First incubation: Place each test strip in an empty channel. Fill each channel with 1.5 mL of the diluted serum sample (1:101 with sample buffer). Incubate for 30 min at room temperature (18–25 °C) on a rocking shaker.
2. Wash: Aspirate off the liquid from each channel and wash 3 × 5 min each with 1.5 mL working-strength wash buffer on a rocking shaker.
3. Second incubation: Pipette 1.5 mL diluted enzyme conjugate (alkaline phosphatase-labelled anti-human IgG) into each channel. Incubate for 30 min at room temperature on a rocking shaker.
4. Wash: Wash as described above.
5. Third incubation: Pipette 1.5 mL substrate solution into the channels of the incubation tray. Incubate for 10 min at room temperature on a rocking shaker.
6. Stop: Aspirate off the liquid from each channel and wash each strip 3 × 1 min with distilled water.
7. Evaluate: Place test strip on the evaluation protocol, air dry and evaluate.

The results of the solid-phase assays for specific autoantibodies were defined as positive with a EUROLineScan Flatbed scanner signal intensity ≥11 according to the EUROIMMUN recommendations, i.e., 11–25 as positive (+), 26–50 as positive (++), and >50 as strong positive (+++); the negative result was defined by a EUROLineScan Flatbed scanner signal intensity ≤10. EUROLINE ANA Profile 23 test kit sorted the autoantibodies of the IgG into the 23 different antigens: dsDNA, nucleosomes, histones, SS-A, Ro-52, SS-B, nRNP/Sm, Sm, Mi-2α, Mi-2β, Ku, CENP A, CENP B, Sp100, PML, Scl-70, PM-Scl100, PM-Scl75, RP11, RP155, gp210, PCNA, and DFS70 in serum. EUROLINE ANA Profile 3 plus DFS70 test kit provided determination of autoantibodies of the IgG for 16 different antigens: nRNP, Sm, SS-A, Ro-52, SS-B, Scl-70, PM-Scl, Jo-1, CENP B, PCNA, dsDNA, nucleosomes, histones, ribosomal P-proteins, AMA-M2, and DFS70 in serum. In addition, to focus on patients who received ANA testing owing to clinical indications, we performed subgroup analysis to exclude samples from subjects for health examination.

### 2.4. Guidance and Re-Evaluation

**Figure 1:**
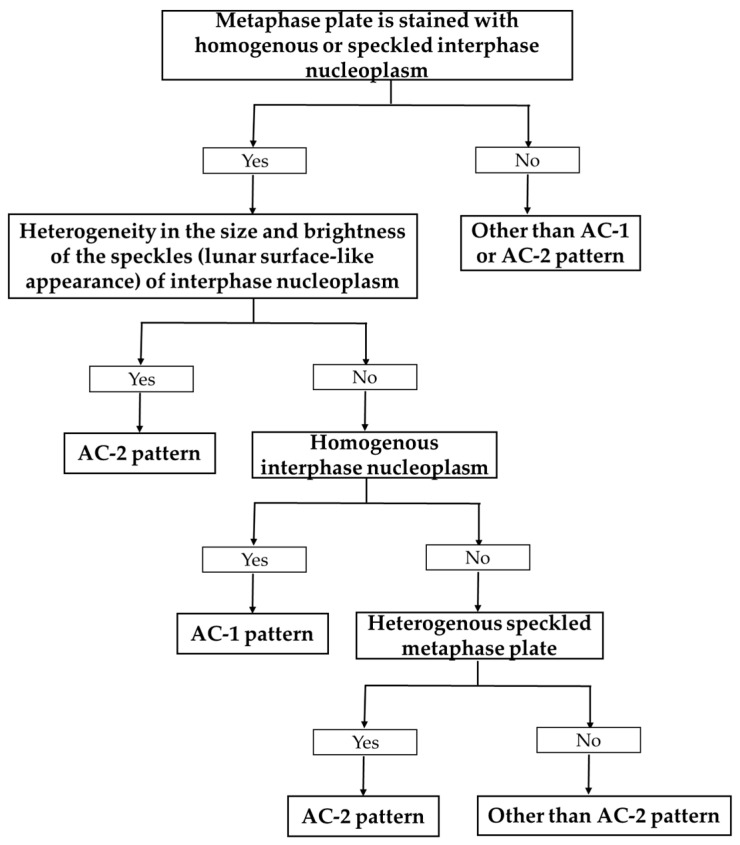
Guidance flowchart to re-evaluate AC-1/AC-2 ANA patterns.

Two rheumatologists and two medical technologists from Linkou CGMH discussed the guidance for discrimination between AC-1/AC-2 ANA patterns. In the event of a disagreement, discussions with the senior rheumatologist (K.-H.Y.) were conducted for a final judgment. After several rounds of discussion, a consensus was reached. Figure 1 shows the final flowchart of the guidance for discrimination between AC-1/AC-2 ANA patterns. Then re-evaluation of the samples using the flowchart as a guide was performed by two rheumatologists. There were three immunofluorescence images of each ANA sample (a total of 186 images), and each image was comprehensively re-evaluated.

### 2.5. Clinical Relevance

Medical records of the included 19 subjects for health examination and 43 patients in CGMH were reviewed to clarify the clinical relationship between ANA patterns and SARDs.

### 2.6. Statistical Analysis

A two-sided *p*-value of less than 0.05 was considered statistically significant. Statistical analyses were performed using the MedCalc Software Ltd. Diagnostic test evaluation calculator, https://www.medcalc.org/calc/diagnostic_test.php (Version 22.013; accessed on 26 September 2023); the MedCalc Software Ltd. odds ratio calculator, https://www.medcalc.org/calc/odds_ratio.php (Version 22.013; accessed on 26 September 2023); and the MedCalc Software Ltd. Two-way Chi-squared test, https://www.medcalc.org/calc/chisquared-2way.php (Version 22.014; accessed on 4 November 2023).

## 3. Results

In the flowchart (Figure 1) for guidance to re-evaluate AC-1/AC-2 ANA patterns, the patterns should not be initially discriminated using a metaphase plate. Instead, the interphase should be inspected first to determine whether it corresponds to the heterogenous or homogenous nucleoplasm pattern. Heterogeneity in the size and brightness of the speckles (we called it “lunar surface-like appearance”) of the interphase nucleoplasm implies an AC-2 pattern, whereas a homogenous interphase nucleoplasm suggests an AC-1 pattern. If the characteristics of the nucleoplasm are ambiguous, the metaphase plate should be used to assign the AC-2 pattern when a heterogenous speckled metaphase plate is present.

Among the total samples, including subjects for health examination and patients, the comparison of the re-evaluated data with the original data showed an increased odds ratio (OR) of detection of anti-nucleosome/histone/dsDNA using AC-1 (from 0.82 to 1.23). In contrast, the comparison of the data excluding 1:80 of ANA endpoint titer with the original data showed a decreased OR of detection of anti-nucleosome/histone/dsDNA using AC-1 (from 0.82 to 0.75). In the subgroup analysis of patient samples only (excluding subjects for health examination), the OR of detection of anti-nucleosome/histone/dsDNA using AC-1 in the original data was 1.36 (95% confidence interval, CI 0.33–5.61, *p*-value = 0.67); furthermore, the OR of detection of anti-nucleosome/histone/dsDNA using AC-1 in the re-evaluated data increased to 5.43 (95% CI 1.00–29.61, *p*-value = 0.05), which was near statistical significance. The data are shown in Table 1.

Concerning the detection ability of AC-2 among total samples, the comparison of the re-evaluated data with the original data showed an increased OR of detection of anti-DFS70 using AC-2 from 4.16 (95% CI 1.39–12.50, *p*-value = 0.01) to 101.86 (95% CI 11.70–886.44, *p*-value < 0.001). However, the comparison of the data, excluding 1:80 of ANA endpoint titer, with the original data showed a decreased OR of detection of anti-DFS70 using AC-2 (from 4.16 to 3.77). In the subgroup analysis for patient samples only, the OR of detection of anti-DFS70 using AC-2 in the original data was 1.89 (95% CI 0.52–6.87, *p*-value = 0.33); in contrast, the OR of detection of anti-DFS70 using AC-2 in re-evaluated data increased to 53.83 (95% CI 5.87–493.61, *p*-value < 0.001). Although a marked improvement in the ability to detect anti-DFS70 using AC-2 was observed after re-evaluation of both total samples and patient samples, a certain number of misclassified samples remained. The false negative rates in total samples and patient samples were 23.3% (7/30) and 26.1% (6/23), respectively. The data are shown in Table 2.

**Figure 2:**
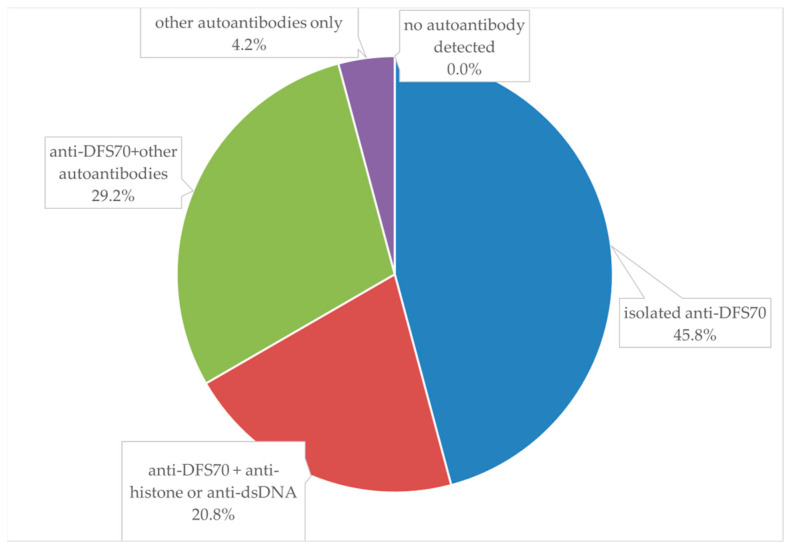
Pie chart of the distribution of isolated anti-DFS70 and anti-DFS70 concomitant with other autoantibodies among re-evaluated total samples with the AC-2 pattern.

Figure 2 shows a pie chart of the distribution of isolated anti-DFS70 and anti-DFS70 concomitant with other autoantibodies among re-evaluated total samples with the AC-2 pattern (24 samples). Anti-DFS70 accounted for 95.8% of the samples with the AC-2 pattern. Nevertheless, only close to half of the samples with the AC-2 pattern (45.8%) had isolated anti-DFS70, and more than half (54.2%) had concomitant specific autoantibodies other than anti-DFS70. The detected specific autoantibodies other than anti-DFS70 are shown in Table 3.

To clarify the relationship between ANA patterns and SARDs, medical records in CGMH for the diagnoses of the included 62 samples were analyzed. None of the 19 subjects for health examination had SARDs, according to their medical records. In contrast, among the 43 patients, there were a total of 10 patients with SARDs, including Sjögren’s syndrome, rheumatoid arthritis, SLE, and ankylosing spondylitis. The specific SARDs, ANA patterns, and detected specific autoantibodies of the 10 patients are listed in Table 4. The percentage of SARDs among patients with ANA pattern AC-1 was 31.8% (7/22), which was higher compared to that of ANA pattern AC-2 (2/18 = 11.1%) not statistically significant (*p*-value = 0.123).

## 4. Discussion

This retrospective study of ANA immunofluorescence imaging revealed a clear improvement in the ability to detect both AC-1 and AC-2 ANA patterns for specific autoantibodies after re-evaluation guided by our flowchart. The difficulty in discriminating between AC-1 and AC-2 may be of concern to the staff of many clinical laboratories. According to our guidance flowchart for re-evaluation, the interphase should first be inspected to determine whether it corresponds to heterogenous or homogenous nucleoplasm, instead of initially discriminating between the patterns based on the metaphase plate. The metaphase plate should be used to make decisions only when there is ambiguity in the characteristics of the nucleoplasm. Our rationale is based on concentrating on the nucleoplasm presentation in the interface since the presentation of the metaphase plate is somewhat perplexing in our experience, as in the case of the AC-1 pattern, which occasionally does not manifest in a homogeneous hyaline fashion in the standard imaging. By prioritizing this focus, the flowchart may resolve the difficult problem for laboratory staff of choosing the nucleoplasm in either the interphase or the metaphase plate in the first step. Our results indicate the value of the flowchart in clinical applications owing to the statistically significant ORs of detection of anti-DFS70 using AC-2 after re-evaluation of the total number of samples (OR 101.9, 95% CI 11.7–886.4, *p*-value < 0.001) and subgroup analysis of the patient samples (OR 53.8, 95% CI 5.9–493.6, *p*-value < 0.001). Although a previous study suggested little need to check the presence of anti-DFS70 [16] and our results revealed an improvement in the ability to detect AC-2 after re-evaluation, there were still a few mistakenly classified samples (a false negative rate of 23.3% in total samples and 26.1% in patient samples). Consequently, solid-phase assays for autoantibodies, including anti-DFS70, may still be indispensable in some clinical situations [17,18].

A retrospective study from Korean university-affiliated hospitals extracted data from 94,153 patients with ANA testing from 2010 to 2019. ANA-associated SARDs were diagnosed in only 0.69% among 94,153 patients and 4.74% among patients testing positive for ANA. The results suggested a need for more careful consideration of when to order ANA testing in clinical practice in order to avoid unnecessary health costs [19]. As a result, considering the controversial role of ANA testing in healthy subjects [20,21], we performed subgroup analysis of patient samples only, and the results still revealed an improvement in detection ability when comparing re-evaluated data with original data, indicating the robustness of our re-evaluation.

In this study, the ORs of detection of anti-nucleosome/histone/dsDNA using AC-1 and anti-DFS70 using AC-2 did not improve after excluding samples with 1:80 of ANA endpoint titer. The normal population has positive ANA results in only 13.3% of cases at titer 1:80 [15] and only 14.0% at titer > 1:100 [22], and screening titers were reported as 1:80 in most laboratories worldwide [14]. In addition, the ANA endpoint titer of at least 1:80 represented the entry criteria of the 2019 European League Against Rheumatism/American College of Rheumatology classification criteria for SLE [6,23]. As a result, the ANA endpoint titer of 1:80 may still hold clinical significance. In most cases, it may be more appropriate to present the ANA data of reports in which the ANA endpoint titers are 1:80 instead of presenting them only as negative results, and physicians can decide its significance based on the patients’ clinical conditions.

Analysis for specific autoantibodies in our study revealed that anti-DFS70 accounted for 95.8% of samples with the AC-2 pattern, which was compatible with the high OR of AC-2 to detect anti-DFS70. However, only close to half of the sample with the AC-2 pattern (45.8%) had isolated anti-DFS70, and more than half of the samples with the AC-2 pattern (54.2%) had concomitant specific autoantibodies other than anti-DFS70. Compared to a previous study [24], our detection rate of concomitant specific autoantibodies was higher, and the possible explanation for this may be our definition of a positive result (including+, ++, and +++). Consequently, it should be emphasized that SARDs with an AC-2 pattern still could not be neglected according to our results. Only when there is isolated anti-DFS70 without other concomitant autoantibodies can the AC-2 pattern be considered a marker of a healthy individual. Although previous studies suggested that an isolated anti-DFS70 ANA could not exclude SARDs [12,18], anti-DFS70 may represent a valuable novel biomarker to improve the interpretation of positive ANAs in samples with negative results for other autoantibodies [25].

The prevalence of the AC-2 pattern was reported to be between 0.3 and 27.0% in patients with ANA detection. In a large ANA-positive cohort, the prevalence of the AC-2 pattern accounted for 3.1%, and 97.6% of the positive AC-2 pattern cases displayed a low ANA titer of (≤1:320) [26]. An increasing trend over time of prevalence of anti-DFS70 autoantibodies was observed among the US population [27]. Although anti-DFS70 autoantibodies are regarded as negatively associated with SARDs, their pathologic or protective influences on diseases remain uncertain [27]. DFS70 plays important roles in the transcriptional activation of specific genes, formation of transcription complexes in active chromatin, DNA repair, regulation of mRNA splicing, and cellular survival against stress, and the biological and clinical significance of anti-DFS70 autoantibodies remains mysterious [8].

The recommendations of ICAP mention ANA patterns and their clinical relevance. AC-1 has been found in patients with SLE, juvenile idiopathic arthritis or chronic autoimmune hepatitis, whereas AC-2 has been commonly found in patients without SARDs or in healthy individuals [1]. Our study showed the higher prevalence of SARDs among patients with the AC-1 ANA pattern compared with those with the AC-2 pattern (31.8% vs. 11.1%). Statistical significance was not demonstrated, potentially due to the small number of the patients. As mentioned earlier, the AC-1 pattern is a homogeneous pattern which is mostly related to SLE. The relationship between homogeneous ANA patterns and related SARDs has been investigated in previous studies. The IIFA for ANA patterns is recognized as a sensitive screening tool rather than a specific test. A retrospective analysis showed that, among 206 patients reactive for specific antibodies, 195 patients (95%) tested positive for ANAs. Within these cases, speckled and homogeneous were identified as the predominant ANA patterns [28]. A cross-sectional study among 495 SLE patients revealed that the most frequently observed ANA patterns were speckled (52.1%) and homogeneous (35.2%) patterns [29]. Another retrospective study with 3510 samples, including 2034 SLE, 973 rheumatoid arthritis, 155 systemic sclerosis, 309 primary Sjögren’s syndrome, and 39 mixed connective tissue disease cases, demonstrated that AC-4 (31.2%) was the main pattern among patients with SARDs, followed by AC-5 (23.9%) and AC-1 (18.8%). SLE predominantly presented with AC-4 (30.3%), followed by AC-5 (26.4%) and AC-1 (20.4%). Primary Sjögren’s syndrome also mostly presented with AC-4 (50.2%), followed by AC-1 (11.0%) and AC-5 (10.4%). Among healthy individuals, 12.2% had positive ANAs (if defined as titer ≥1:100), and the major ANA pattern was AC-2 [30]. Despite being considered a screening tool for SARDs, it is important to note that ANA testing is less sensitive for certain SARDs, including Sjögren’s syndrome and idiopathic inflammatory myopathy [31].

Our study has some limitations. First, the retrieved ANA samples were relatively few. Our study may be considered as a pilot study for discrimination between ANA AC-1 and AC-2 patterns. Further studies with more ANA samples are required to ascertain the robustness of our flowchart and to clarify the relationship between ANA patterns and specific SARDs. Second, we analyzed the data with and without healthy subjects. Considering that ANAs could occur in subjects with a diversity of other conditions and in healthy subjects, ANA testing may result in false-positive results [31]. Consequently, traditional guidelines suggest ANA testing only in individuals with clinical suspicion of rheumatic conditions [20]. However, positive ANAs may be associated with immune dysfunction and several non-autoimmune diseases, and routine ANA testing for healthy people has been suggested [10,21,32]. As a result, the application of ANA testing in health examinations remains controversial and needs further investigation.

## 5. Conclusions

The flowchart to discriminate between AC-1 and AC-2 ANA patterns in this study offers a practical guidance for other laboratories when encountering ambiguous results. The ability to detect anti-nucleosome/histone/dsDNA using the AC-1 ANA pattern and of anti-DFS70 using the AC-2 ANA pattern demonstrated improvement after re-evaluation, especially that of anti-DFS70 using the AC-2 ANA pattern. Further studies with more ANA samples may be required to confirm our results.

## Figures and Tables

**Table 1:** Detection of anti-nucleosome/histone/dsDNA using AC-1 ANA pattern.

	Anti-Nucleosome/ Histone/dsDNA (+) *	Anti-Nucleosome/ Histone/dsDNA (−)	LR+/LR−	OR (95% CI)	*p*-Value
Total (healthy subjects + patients)—Original					
AC-1 (+)	6	23	0.89/1.10	0.82 (0.25–2.71)	0.74
AC-1 (−)	8	25			
Total (healthy subjects + patients)—Excluding 1:80					
AC-1 (+)	5	19	0.86/1.14	0.75 (0.20–2.78)	0.67
AC-1 (−)	7	20			
Total (healthy subjects + patients)—Re-evaluated					
AC-1 (+)	8	25	1.10/0.89	1.23 (0.37–4.07)	0.74
AC-1 (−)	6	23			
Patients—Original					
AC-1 (+)	5	14	1.18/0.87	1.36 (0.33–5.61)	0.67
AC-1 (−)	5	19			
Patients—Re-evaluated					
AC-1 (+)	8	14	1.89/0.35	5.43 (1.00–29.61)	0.05
AC-1 (−)	2	19			

LR, likelihood ratio; OR, odds ratio; CI, confidence interval. * At least one of the positive results of three autoantibodies: anti-nucleosome, anti-histone, or anti-dsDNA.

**Table 2:** Detection of anti-DFS70 using the AC-2 ANA pattern.

	Anti-DFS70 (+)	Anti-DFS70 (−)	LR+/LR−	OR (95% CI)	*p*-Value
Total (healthy subjects + patients)—Original					
AC-2 (+)	24	14	1.71/0.41	4.16 (1.39–12.50)	0.01
AC-2 (−)	7	17			
Total (healthy subjects + patients)—Excluded 1:80					
AC-2 (+)	22	10	1.67/0.44	3.77 (1.14–12.46)	0.03
AC-2 (−)	7	12			
Total (healthy subjects + patients)—Re-evaluated					
AC-2 (+)	23	1	24.53/0.24	101.86 (11.70–886.44)	<0.001
AC-2 (−)	7	31			
Patients—Original					
AC-2 (+)	17	12	1.23/0.65	1.89 (0.52–6.87)	0.33
AC-2 (−)	6	8			
Patients—Re-evaluated					
AC-2 (+)	17	1	14.78/0.27	53.83 (5.87–493.61)	<0.001
AC-2 (−)	6	19			

LR, likelihood ratio; OR, odds ratio; CI, confidence interval.

**Table 3:** Detection of specific autoantibodies (other than anti-DFS70) in 24 samples with the AC-2 ANA pattern.

	*n*	Percentage
Specific autoantibodies (+)/AC-2	13/24	54.2% *
dsDNA	4	16.7%
histone	2	8.3%
Sm	1	4.2%
RNP/Sm	2	8.3%
RP155	4	16.7%
Ku	1	4.2%
PM-Scl75	3	12.5%
Sp100	1	4.2%
Mi-2a	1	4.2%
Mi-2b	1	4.2%

RP155, recombinant subunit POLR3A of human RNA polymerase III. * Sum of the percentage of each specific autoantibody is more than 54.2% because each AC-2 pattern may be detected with more than one autoantibody.

**Table 4:** The 10 patients with systemic autoimmune rheumatic diseases (SARDs) among the total 43 patients.

	*n*	ANA Pattern (Re-Evaluated)	Specific Autoantibodies
SARDs (+)/patients	10 */43		
Sjögren’s syndrome	5	AC-1	AMA-M2, histone, nucleosome, dsDNA, SSB, Ro52, SSA
		AC-1 & AC-4	Ro-52, SSA, histone, DFS70
		AC-1	nil
		AC-1	RP155, PM100, dsDNA
		AC-1	RP155, Ro-52, SSA
RA	3	AC-1	nil
		AC-1	RP11, RP155
		AC-2	PM75, Sp100, Mi-2b, DFS70
SLE	2	AC-1	AMA-M2, histone, nucleosome, dsDNA, SSB, Ro52, SSA
		AC-4	Ku, SSB, Ro-52, SSA
AS	1	AC-2	RP-155

RP155, recombinant subunit POLR3A of human RNA polymerase III; RP11, recombinant subunit POLR3K of human RNA polymerase III; RA, rheumatoid arthritis; SLE, systemic lupus erythematosus; AS, ankylosing spondylitis. * Sum of the patient number with each SARD is more than 10 because one of the 10 patients with SARDs is diagnosed as having both Sjögren’s syndrome and SLE.

## Data Availability

Data are contained within the article.
